# Perceived Emotional Intelligence and Learning Strategies in Spanish University Students: A New Perspective from a Canonical Non-symmetrical Correspondence Analysis

**DOI:** 10.3389/fpsyg.2017.01888

**Published:** 2017-10-27

**Authors:** María C. Vega-Hernández, María C. Patino-Alonso, Rosario Cabello, María P. Galindo-Villardón, Pablo Fernández-Berrocal

**Affiliations:** ^1^Department of Statistics, University of Salamanca, Salamanca, Spain; ^2^Institute of Biomedical Research of Salamanca, Salamanca, Spain; ^3^Department of Developmental and Educational Psychology, University of Granada, Granada, Spain; ^4^Department of Basic Psychology, Faculty of Psychology, University of Málaga, Málaga, Spain

**Keywords:** perceived emotional intelligence, learning strategies, canonical non-symmetrical correspondence analysis, university students, gender

## Abstract

Recent studies have revealed that emotional competences are relevant to the student’s learning process and, more specifically, in the use of learning strategies (LSs). The aim of this study is twofold. First, we aim to analyze the relationship between perceived emotional intelligence (PEI) and LSs applying the scales TMMS-24 and Abridged ACRA to a sample of 2334 Spanish university students, whilst also exploring possible gender differences. Second, we aim to propose a methodological alternative based on the Canonical non-symmetrical correspondence analysis (CNCA), as an alternative to the methods traditionally used in Psychology and Education. Our results show that PEI has an impact on the LS of the students. Male participants with high scores on learning support strategies are positively related to high attention, clarity, and emotional repair. However, the use of cognitive and control LS is related to low values on the PEI dimensions. For women, high scores on cognitive, control, and learning support LS are related to high emotional attention, whereas dimensions such as study habits and learning support are related to adequate emotional repair. Participants in the 18–19 and 22–23 years age groups showed similar behavior. High scores on learning support strategies are related to high values on three dimensions of the PEI, and high values of study habits show high values for clarity and low values for attention and repair. The 20–21 and older than 24 years age groups behaved similarly. High scores on learning support strategies are related to low values on clarity, and study habits show high values for clarity and repair. This article presents the relationship between PEI and LS in university students, the differences by gender and age, and CNCA as an alternative method to techniques used in this field to study this association.

## Introduction

Over the last few years, university education has been facing important changes that affect both students and lecturers. New study plans based on the ECTS system (European Credit Transfer System) have been implemented (Real Decreto 1125/2003), which means that the autonomous working time of the students has to be taken into account. The implementation of the European Higher Education Area (EHEA) has entailed a transformation of higher education in Spain. Further, these changes have generated controversy among both lecturers and students, as the learning plans have been drastically changed (Comisión Europea, 2004; Quevedo-Blasco et al., 2015). Hence, university didactic has changed from a system focused on the lecturer, to a system that focuses on the students, thus forcing the students to acquire a relevant role in the teaching-learning process. One of the implications is that lecturing, studying, tutorials, seminars, essays, practical classes, or preparation for exams and evaluations are timed. This system implies that the students need to acquire new forms of learning and developing learning strategies (LSs). For this reason, it is important to know the variables that intervene in the learning of students of higher education and the possible relationships that exist between them. Thus, teachers will be able to improve their didactic methodologies to help students to learn more effectively.

There is a large body of evidence pointing to the important role of LS in predicting academic achievement in college (e.g., Richardson et al., 2012). An inclusive definition of LS regards these strategies as control mechanisms that guide an individual’s information processing, facilitating the acquisition, storage, and recovery of information (Clemente et al., 1992; Bahamón et al., 2012; Richardson et al., 2012). The students construct their knowledge using tools that favor self-learning, guaranteeing more efficient studying time and decreasing the likelihood of academic failure (Beltrán, 1993; Petrides et al., 2004). In the educational context, poor use of LS has a negative impact on the student’s personal development, causing them to avoid studying those concepts that are more difficult, which is detrimental to learning (Richardson et al., 2012; Ruffing et al., 2015).

Students experience emotions they cannot always control. These affective reactions that appear suddenly in response to a characteristic situation or a stimulus, predispose the individual to a number of different biological consequences that must be considered in the learning process. Put simply, when a student is determined to solve a problem and is successful then they will experience positive emotions and feelings, whereas if they fail they will show negative emotions. Given that students play the main role in higher education, the influence of emotions and feelings is of great interest in academic performance. Many of the competences included in the studied subjects refer to aspects related to the emotional and social development of the students. Although the programs do not allude to the development of emotional competences, the intention is to educate people to increase their capacity to adapt to any circumstance in the present society, which should include social and emotional training. In this regard, Mayer and Salovey published the first article related to the issue of emotional intelligence (EI). Since then, scientific evidence has shown how EI is linked to different positive indicators of the human being in general and in higher education in particular (Salovey and Mayer, 1989; Mayer and Salovey, 1993, 1997; Parker et al., 2004).

Emotional intelligence, following the model of Mayer and Salovey (1997, p. 10), can be defined as *the ability to perceive accurately, appraise and express emotion; the ability to access and/or generate feelings when they facilitate thought; the ability to understand emotion and emotional knowledge; and the ability to regulate emotions to promote emotional and intellectual growth*. According to this model, EI involves a set of abilities related to the emotional processing of information. More specifically, EI is part of a model with four interrelated branches: emotional perception and expression, emotional facilitation, emotional comprehension, and regulation (Mayer et al., 2016). In the university context, this concept is related to complementary aptitudes that are different from academic intelligence or purely cognitive abilities, but have a great impact on the general development of the students (Costa and Faria, 2015; Nathanson et al., 2016). This construct has been evaluated with execution and self-reporting scales, depending on which of the theoretical models of EI is used (Matthews et al., 2004; Mayer et al., 2008). For our study, we will use the Trait Meta-Mood Scale (TMMS) (Salovey et al., 1995), which evaluates EI in accordance with the ability model developed by Mayer and Salovey (1997). More specifically, we have used the “Trait Meta-Mood Scale-24” (TMMS-24) of Fernández-Berrocal et al. (2004), which evaluates EI using self-reports and provides the subjective perception of the participants about their own EI in Spanish speaking populations. In particular, the TMMS-24 provides information about perceived emotional intelligence (PEI) by evaluating three dimensions: emotional attention, emotional clarity, and emotional regulation. However, the EI has shown to be beneficial for a number of relevant variables related to the educational context. To be more specific, EI is related to different variables such as better physical and mental health (Martins et al., 2010; Fernández-Berrocal and Extremera, 2016), better well-being, and less substance consumption (Fernández-Berrocal and Extremera, 2009; Sánchez-Álvarez et al., 2016; Serrano and Vaillo, 2016), less aggressive behaviors (García-Sancho et al., 2014), as well as better academic performance (Lanciano and Curci, 2014; Costa and Faria, 2015).

The presence of emotional abilities or competences in the academic context is relevant given its impact on the general development of the students (Costa et al., 2013; González et al., 2013; Nathanson et al., 2016). Several studies have suggested that academic performance is better if the students have emotional skills and are more actively involved in their own education (Schutte et al., 2001; Barchard, 2003; Durlak et al., 2011; Gutiérrez-Cobo et al., 2017). Further, some studies suggest that women show better EI abilities throughout life and that these gender differences have an impact on both personal and academic aspects of life (Brackett and Mayer, 2003; Cabello and Fernández-Berrocal, 2015; Cabello et al., 2016).

Relatively little research, however, has been carried out to analyze the relationship between PEI and LS. A number of studies with Iranian university students from different fields of study have found that students (both male and female) that are emotionally intelligent use more LS (Hasanzadeh and Shahmohamadi, 2011; Zafari and Biria, 2014; Soodmand et al., 2016). However, some studies concerning the relationship between PEI and LS with Spanish speaking participants have found a more complex pattern of results. García-Fernández et al. (2015), using a sample of 1253 Chilean students, analyzed the role of EI in the educational context and its influence on LS using TMMS-24 (Fernández-Berrocal et al., 2004) and the Inventory of Learning and Study Strategies – High School version (Weinstein and Palmer, 1990). These authors identified four distinct profiles according to PEI (HGEI: high EI; LAHR: low attention and high emotional repair; HALCR: high attention and low emotional clarity; LGEI: low EI). They also found that young participants with HGEI or LAHR used more LS than those with HALCR or LGEI. In this regard, Inglés et al. (2017) studied PEI and LS with 1071 Spanish secondary school students (aged between 14 and 17 years) using the TMMS-24 and the Learning Strategies Questionnaire (LSQ) (Beltrán et al., 2006). They identified four profiles of PEI according to the combinations of its dimensions (GLEI: low EI; HALR: high attention and low emotional repair; GHEI: high EI; LAHR: low attention and high emotional repair). They found that the students with profiles GHEI and LAHR used more LS than those students with profiles GLEI or HALR, that is, those students with high scores on PEI and those that presented low attention and high emotional repair used more LS that the other groups of students.

Conventional methods of analysis for the study of relationships between variables are usually correlation or regression analyses (Balluerka et al., 2013; Cejudo et al., 2016). Nevertheless, sometimes problems with data can generate confusion in the interpretation of analyses. For this reason, we present an alternative statistical procedure that complements the traditional techniques, known as the Canonical non-symmetrical correspondence analysis (CNCA) (Willems and Galindo-Villardon, 2008).

The present study had three main objectives: (1) to analyze the relation between PEI and LS in a sample of Spanish university students, (2) to explore the possible gender differences in the relationship between PEI and LS, and (3) to propose an alternative method to the traditionally used statistical analyses such as regression in the Psychology and Education context, that is, the use of the CNCA.

We propose the following hypotheses: (1) due to the fact that there are differences in the PEI associated with gender we assume that the relationship between LS and PEI will be different among Spanish men and women at university, (2) men with high levels of PEI will make more use of LS, (3) women with high levels of emotional attention will use cognitive and control strategies and learning support, and (4) the multivariate CNCA methodology will yield more stable results than the techniques traditionally used such as regression analysis in the field of Psychology and Education.

## Materials and Methods

### Participants

The sample consisted of 2334 Spanish students from the University of Salamanca (USAL), divided into five age groups: 18–19 years (51.4%), 20–21 years (24.9%), 22–23 years (14.0%), and 24 years or more (9.8%). The gender distribution of the university students was 36.3% male and 63.7% female participants. Of the whole sample, 10.30% of the students were studying Art and Humanities, 10.9% were studying Science, and 15.9% of the students were studying Engineering and Architecture. The biggest groups were within Health Science (30.2%), and Social and Legal Sciences (32.8%). With respect to their academic year, 39.1% of the sample was in the first year, 32.6% was in the second year, 17.5% was on the third year, and 10.9% in other years.

### Method of Assessment

#### *Trait Meta-Mood Scale-24* (TMMS-24) (Fernández-Berrocal et al., 2004)

This instrument was designed to assess how people reflect upon their moods and provides an indicator of the levels of perceived EI. Respondents are asked to rate their degree of agreement on each of the 24 items on a five-point Likert-type scale ranging from 1 (very much agree) to 5 (very much disagree). The scale is composed of three sub-factors: Attention to one’s Feelings, Emotional Clarity, and Mood Repair. Attention to Feelings, assessed by the first eight items, is the degree to which people believe they pay attention to their own feelings (i.e., “I think about my mood constantly”); Emotional Clarity, evaluated by the following eight items, refers to how people believe that they perceive their emotions (i.e., “I frequently make mistakes about my feelings”), and Mood Repair, assessed by the remaining eight items, refers to people’s belief in their capacity to block negative moods and prolong positive moods (i.e., “Although I sometimes feel sad, I usually have an optimistic outlook”). Fernández-Berrocal et al. (2004) found an internal consistency of 0.90 for Attention, 0.90 for Clarity, and 0.86 for Repair, improving the psychometric properties of the original extended 48-item version (Salovey et al., 1995), which had values of 0.86, 0.87, and 0.82 for Attention, Clarity, and Repair, respectively.

#### *Abridged ACRA Scale of Learning Strategies* for College Students (De la Fuente and Justicia, 2003)

The ACRA scale takes its abbreviated name based on an adaptation from the original (the Learning Strategies Scale, ACRA) (Román and Gallego, 1994), which focuses on high school students. It includes 44 items with a Likert scale with four possible answers. However, the distribution of the responses shows that it would be convenient to use the information by grouping the answers few-nothing and enough-a lot together. It has been designed to include information about LSs and techniques in the university population (Justicia and De la Fuente, 1999; De la Fuente and Justicia, 2003; Gargallo et al., 2007). Abridged ACRA is divided into three dimensions: Cognitive and control learning strategies, Learning support strategies, and Study habits. The dimension of Cognitive and control learning strategies includes 25 items that refer to selection, organization, highlighting, awareness of the functionality of the strategies, elaboration strategies, planning and control of the answer during evaluation, repetition, and re-reading (i.e., “I produce summaries with the help of words or phrases previously underlined”). The dimension Support Strategies includes 14 motivational and affective items, such as intrinsic motivation, anxiety control, non-distraction conditions, need for social support, timing, and scheduling (i.e., “I study in order to broaden my knowledge, to know more, in order to be more expert”). Finally, the Study Habits dimension includes five items that involve understanding and study habits (i.e., “I try to express what I have learned in my own words, instead of repeating literally what the teacher or the book says”).

### Procedure

The research data were collected using questionnaires, and the confidentiality and anonymity of the participants was guaranteed. The study was carried out in accordance with the Declaration of Helsinki and ethical guidelines of the American Psychological Association, and all the participants signed a consent form prior to their participation in the study. The Research Ethics Committee of the University of Málaga approved the study protocol as part of the project SEJ-07325.

We used a multi-stage sampling method. In the first stage we used a stratified probabilistic sampling method according to the different knowledge areas (Arts and Humanities, Science, Health Sciences, Social and Legal Sciences, and Architecture). In the second stage we used a simple random sampling method for each stratum.

### Statistical Analysis

We used a unidimensional descriptive study with each of the dimensions of the PEI evaluated by the TMMS-24 along with the LS used by the students. Quantitative variables were expressed as mean ± standard deviation (SD). We tested the reliability of questionnaires through the assessment of internal consistency with the use of Cronbach’s Alpha, McDonald’s Omega, and Greatest Lower Bound (GLB) coefficients. We used Cronbach’s Alpha because it is the most widely used method for estimating internal consistency reliability. However, this procedure has limitations, and for this reason, we use McDonald’s Omega and GLB coefficients, which provide better results when the items are Likert-type and have skewed distributions. The first considers commonality and the second uses factorial procedures such as minimum rank factor analysis, but more recently the GLBalgebraic (GLBa) procedure has been developed from an algorithm that introduces a vector to weight the items by importance. The differences between the quantitative variables of the two categories were analyzed with non-parametric Mann–Whitney *U*-test for independent samples and in the case of more than two groups we used the Kruskal–Wallis test.

In Psychology and Education, the traditional ways to analyze relationships between studied variables are correlation and regression analyses (Moreno et al., 2006; Bazán-Ramírez et al., 2011; De Haro and Castejón, 2014; Azpiazu et al., 2015). However, it is sometimes the case that the distribution is not homogeneous and we can find a modal category or even the Simpson paradox (Simpson, 1951). The Simpson paradox can lead to error in the interpretation of the results, produce a change in the relationship between the variables, and even change the direction of the relationship between the variables when the sample is divided into sub-samples (Malinas and Bigelow, 2009). Moreover, we can decrease the multiple correlations by increasing the simple correlations. It should also be noted that we need to take into account the concept “statistical significance,” which depends on two basic elements: the magnitude of the difference that we wish to measure, and the size of the sample. The size of the sample affects statistical significance through the standard error (which becomes smaller as we include more participants in the sample), so any difference between the variables can be statistically significant if we have access to a sample that is sufficiently large. Thus, we can find statistically significant differences and small coefficients, even close to zero, that cannot capture the actual direction of the relationship, which could be reflected on dot clouds without any linearity.

In our current work, we present an alternative statistical procedure to the traditional techniques such as regression analysis. In order to explore our results obtained by both procedures, and to analyze whether the dimensions of PEI are related to the LS scales used by the students, we carried out a series of hierarchical regression analyses. We first included gender and age as co-variables. We then introduced the dimensions of the TMMS-24 as the predictive variables. We then decided to use an alternative estimation method, the CNCA method, which allows us to study the relationships between PEI and LS as a whole (Willems and Galindo-Villardon, 2008). We began with two matrices of data, one of which contained the information about LS (Cognitive and control learning strategies, Learning Support strategies, and Studying habits) for all 2334 participants. The second matrix contained information about the dimensions of the PEI (Attention, Clarity, and Repair). The CNCA orders the data based on estimated values of the dimensions of the PEI depending on the different LS. CNCA has the advantage that, when we have co-linearity, it allows us to reach the aimed objective and it without affecting the coefficients. Our procedure selects the lineal combination of the dimensions of the PEI that maximizes the dispersion of the values of different LS. Thus, the different LS can be explained through a model in which the explanatory variable is a lineal combination of the dimensions that evaluate the PEI. The results are presented with an ordering diagram where the LS are represented by dots and the dimensions of the PEI are represented by vectors. The angle that the respective items form between them, which evaluate different aspects of the PEI, allows us to estimate the degree of co-variation between the different aspects. Acute angles indicate a strong relationship; straight angles indicate independence between both aspects; and obtuse angles indicate an inverse relationship. To evaluate the relationship between each one of the dimensions of the PEI for each of the LS, we only have to draw a perpendicular line to the vector that links the dimension-PEI and the coordinates origin. The points that represent the different LS, and which projections over the vector dimension-PEI are closer to the end of the arrow are taken to indicate that they have higher percentages with respect to that dimension.

This is the first study to use the CNCA method to obtain a relationship between two instruments within the Psychology and Education contexts. However, prior to the proposal to use the method in this study, the CCA (Canonical Correspondence Analysis) (Ter Braak, 1986) was developed in the vegetation modeling context, and it has also been used within the Psychology context (López et al., 2014; Vicente-Galindo et al., 2017).

The data were analyzed using IBM SPSS Statistics package, Version 23.0 and the cncaGUI R package (Nieto et al., 2015).

## Results

### Descriptive Statistics

**Table 1** shows descriptive analyses of the dimensions of the PEI and LS. Means and standard deviation for the TMMS-24 and Abridged ACRA subscales are shown in **Table 1**. Emotional repair has a greater average with respect to other dimensions of the PEI, and cognitive and learning control strategies has the highest mean value in LS dimensions. Internal consistency for all three TMMS-24 subscales was strong, with Cronbach’s Alpha, Omega, GLB, and GLBa ranging from 0.84 to 0.930. Two subscales of the Abridged ACRA are consistent, but the study habits scale presents weak coefficients that are less than 0.6, because only five items form this subscale (see **Table 1**).

**Table 1:** Descriptive statistics.

	Mean	*SD*	α	ω	GLB	GLBa
**Perceived emotional intelligence**						
Attention	3.08	0.89	0.89	0.89	0.93	0.93
Clarity	3.11	0.84	0.88	0.88	0.91	0.92
Repair	3.28	0.84	0.84	0.85	0.89	0.89
**Learning strategies**						
Cognitive and learning control strategies	0.80	0.15	0.79	0.79	0.81	0.82
Learning support strategies	0.75	0.18	0.66	0.67	0.73	0.75
Study habits	0.74	0.25	0.47	0.52	0.57	0.56

**Table 2** presents the covariance matrix between subscales of TMMS-24 and Abridged ACRA. We observed that the values among the dimensions of PEI are greater than those of LS, emphasizing the covariances between emotional clarity and repair and attention.

**Table 2:** Covariance matrix between subscales of TMMS-24 and Abridged ACRA.

	1	2	3	4	5	6
(1) Cognitive and learning control strategies	0.023					
(2) Learning support strategies	0.015	0.032				
(3) Study habits	0.013	0.012	0.061			
(4) Attention	0.021	0.027	0.009	0.787		
(5) Clarity	0.018	0.025	0.022	0.252	0.708	
(6) Repair	0.018	0.035	0.034	0.146	0.296	0.714

We next present gender differences in the studied variables. In relation to PEI, women show higher attention toward their feelings than men (*Z* = -5.28, *p* < 0.01). We failed to find any significant differences for emotional clarity (*Z* = -0.70, *p* > 0.05) or emotional repair (*Z* = -0.14, *p* > 0.05). With respect to LS, male students reported that they used more learning support strategies (*Z* = -10.22, *p* < 0.01) and study habits (*Z* = -2.31, *p* = 0.02) than female students. However, the female students used more cognitive and control LSs (*Z* = -14.08, *p* < 0.01) (see **Table 3**).

**Table 3:** Descriptive statistics by gender.

	Males *n* = (847)		Females *n* = (1487)			
	Mean	*SD*	Mean	*SD*	*Z*	Significance
**Perceived emotional intelligence**						
Attention	2.95	0.90	3.16	0.87	–5.28	<0.01
Clarity	3.13	0.84	3.09	0.84	–0.70	0.48
Repair	3.27	0.85	3.28	0.84	–0.14	0.89
**Learning strategies**						
Cognitive and learning control strategies	0.74	0.16	0.83	0.13	–14.08	<0.01
Learning support strategies	1.70	0.19	0.78	0.17	–10.22	<0.01
Study habits	1.73	0.24	0.75	0.25	–2.31	0.02

According to age groups, with respect to the PEI, the youngest students (18–19 years) pay more attention to their feelings (Chi-square = 16.36, df = 3, *p* < 0.01), use more cognitive and control strategies (*p* < 0.01), and finally use more learning support strategies (*p* < 0.01).

### Hierarchical Multiple Regression

We explored the effects of PEI dimensions on LSs beyond the effects of age and gender. To this end, we conducted several hierarchical multiple regression analyses where the LSs dimensions were regressed onto age and gender in the first step and PEI dimensions in the final step (see **Table 4**). The dimensions of the LS were analyzed separately, adjusting a model for each of them. The results show that gender and age could explain 9% of the variance for cognitive and control strategies, 5% of the variance for learning support strategies and 1% of the variance for study habits. The addition of the second model of the dimensions of the PEI meant a significant increase of 3% in the explained variance of the learning cognitive and control strategies, 7% for learning support strategies, and 2% for study habits. Thus, these results demonstrate that the students that reported higher levels of use of cognitive and control LS strategies presented a higher perceived ability to understand and regulate their emotions. For the dimension of LS, study habits and emotional attention were not significant.

**Table 4:** Hierarchical multiple regression predicting Learning Strategies.

	*B*	SE_B_	β	*R*^2^	*F*	Δ*R*^2^
**Cognitive and learning control strategies**						
(1) Gender	0.10**	0.006	–0.30	0.09	115.83**	0.09
Age	–0.01+	0.003	–0.03			
(2) Attention	0.01**	0.004	0.07	0.12	65.88**	0.03
Clarity	0.01**	0.004	0.08			
Repair	0.02**	0.004	0.10			
**Learning support strategies**						
(1) Gender	0.08**	0.008	–0.21	0.05	66.69**	0.05
Age	–0.02**	0.004	–0.08			
(2) Attention	0.02**	0.004	0.08	0.12	62.83**	0.07
Clarity	0.01**	0.005	0.07			
Repair	0.04**	0.005	0.19			
**Study habits**						
(1) Gender	0.02+	0.011	–0.04	0.01	2.50+	0.01
Age	–0.01	0.005	–1.02			
(2) Attention	–0.01	0.006	–1.01	0.03	15.01**	0.02
Clarity	0.02*	0.007	1.05			
Repair	0.04**	0.007	0.15			

*^+^*p* < 0.10; ^∗^*p* < 0.05; ^∗∗^*p* < 0.01. Gender (male = 0, female = 1).*

We clearly observed statistically significant results when the part that explains these variables is small, as a consequence of the sample size. Further, the distribution of the scale used to evaluate LS is not homogeneous given that the students generally use LS and hence report this in the questionnaire, although they do not use all the LSs to the same degree, as this depends on gender and age. In order to address these problems, we introduced an alternative statistical procedure, CNCA.

### Multivariate Characterization of the Relationship between Perceived Emotional Intelligence and Learning Strategies

The relationship between PEI and LS is analyzed with a CNCA combined with the dimensions of both instruments (TMMS-24 and Abridged ACRA). **Table 5** represents the variability explained for each axis and the accumulated variability for the projected space, that is, the total inertia explained by the adjusted values. The first two axes explain the total variability, both for male and female participants.

**Table 5:** Values and proportion of inertia explained by each axis in the projected space.

	Explained variability (%)	Accumulated variability (%)
**Females**		
Axis I	88.76	88.76
Axis II	11.24	100.00
**Males**		
Axis I	63.77	63.77
Axis II	36.23	100.00

**FIGURE 1:**
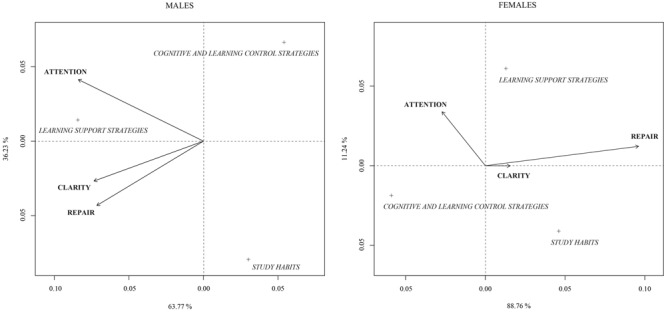
Ordering diagram of the Canonical non-symmetrical analysis between PEI and LS depending on gender.

We will now present the ordering diagram from the CNCA (**Figure 1**).

As described in the Section “Materials and Methods,” the dimensions of the PEI are represented by vectors. Regarding participants, we can highlight the small angle that is formed between the repair and clarity dimensions of the PEI, which indicates that when the students score high on one dimension, they also score high on the other. In the case of female participants, we can observe that clarity is not well-represented due to the short longitude of the vector. However, for the attention and emotional repair dimensions, the graph shows an inverse relationship between both aspects.

When we analyze the dimensions of LS, we observe that both males and females are perfectly differentiated, sharing no similarities, since there is a substantial difference between their points. In order to evaluate the impact that a given item of the PEI has on each of the aspects of LS, we drew a perpendicular line to the vector that links the dimension of the PEI with the coordinates origin of each LS.

For male participants, we observed that the learning support strategies dimension pointed at the higher extreme of the vector for the three dimensions of the PEI. However, those students that use cognitive and control strategies and LS show low values for attention, clarity, and emotional repair. For the female participants, we observed that both cognitive and control strategies and support strategies show high values for emotional attention and low values for study habits, which are related to repair.

**Table 6** shows the information given by the direction of the factorial axis about the LS. Regarding the LS, we observed that those that contribute most to form the first axis are cognitive and control strategies and study habits strategies in the female participants. However, for male participants learning support strategies are the most influential in forming this axis.

**Table 6:** Relative contributions of the first two factors to Learning Strategies.

	Projected space	
	Axis 1	Axis 2
**Females**		
Cognitive and learning control strategies	986.84	13.16
Learning support strategies	263.63	736.37
Study habits	906.83	93.17
**Males**		
Cognitive and learning control strategies	541.69	458.32
Learning support strategies	984.89	15.11
Study habits	200.29	799.71

**FIGURE 2:**
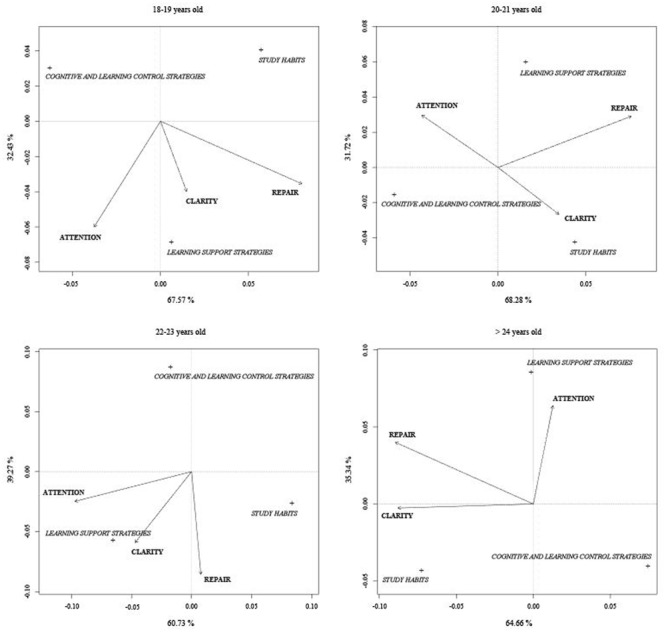
Ordering diagram of the Canonical non-symmetrical analysis between PEI and LS depending on age.

In addition, for the four age groups, the first two axes explain the total variability. Axis I absorbs more than 60% in all cases (67.57% 18–19 years old, 68.28% 20–21 years old, 60.73% 22–23 years old, and 64.66% over 24 years old). We present the ordering diagram from the CNCA for age groups in **Figure 2**.

In addition to the small angle formed between the repair and clarity dimensions of the IEP, the participants aged 18–19 and 22–23 years also present a small angle between the attention and clarity dimensions. For participants aged 18–19 and 22–23 years, we observed that the dimension of learning support strategies pointed to the upper end of the vector for the three dimensions of the IEP. Cognitive and control strategies have low clarity and repair values. Moreover, study habits show high values for emotional clarity and low values for attention and repair. However, those students aged 20–21 years and older than 24 years show high values of study habits in clarity and repair and low values of attention. The dimension of learning support strategies scores high values on attention and repair and low or medium values on clarity. Further, cognitive and control strategies have low scores in the group of participants older than 24 years but high scores in the 20–21 years age group (**Figure 2**).

We observed that the LS that contribute most to form the first axis are cognitive and control strategies and study habits strategies, except for the 22–23 year age group in which cognitive and control strategies exert more influence on the second axis.

## Discussion

This study confirmed the existence of a relationship between PEI and LS in university students. The CNCA analysis revealed that male students with high scores on support strategies also show high attention, clarity, and emotional repair. However, the use of cognitive and control LS is related to low levels of attention, clarity, and emotional repair. For female participants, high scores on cognitive and control LS and support strategies are related to high emotional attention. The 18–19 and 22–23 years age groups showed similar behavior and groups aged 20–21 years and older than 24 years behaved in a similar way.

These results are generally consistent in with other studies using Iranian students that found a positive relationship between EI and LS in different knowledge areas in both male and female samples (Hasanzadeh and Shahmohamadi, 2011; Zafari and Biria, 2014; Soodmand et al., 2016). More specifically, our results a similar to those published by García-Fernández et al. (2015) and Inglés et al. (2017) using Spanish speaking teenagers. These studies identified four different profiles according to PEI. The profiles “high EI” or “low attention and high emotional repair” were those that used more LS compared with the other profiles. Our results are partially consistent with the findings of this study. The participants with high scores for PEI in our study use more learning support strategies (like the students in the described study with “high EI”). This could be due to the fact that this support is also affected by external aspects, such as the way in which we interact with others. However, our results are not consistent with the previously described study for the female participants with the profile “high attention,” given that in our sample, this profile of female participants use more strategies, except those related to study habits.

The most apparent results obtained with both statistical methods are concordant; however, CNCA represents an alternative method to hierarchical multiple regression, and presents results graphically in the form of an ordering diagram that is easy to interpret. In addition, when we have co-linearity, CNCA allows us to reach the aimed objective without affecting the coefficients.

A strength of our study is that we introduced the CNCA as an alternative to the traditional methods of data analysis. CNCA orders data according to the LS used by the students, and this ordering is conditioned by the PEI results of those students. This analysis has the advantage of working in terms of inertia absorption, which is not affected by the sample size and, when one or more variables are conditioning the behavior of another, it shows the asymmetrical features of the information. This technique also allows us to characterize data more precisely since it jointly analyses all the dimensions of the LS rather than using separate analyses as in the regression analysis.

The knowledge gained from the findings of this research implies possible changes in teaching methodologies. In particular, teachers should be able to adapt their teaching on the basis of their knowledge regarding the levels of EI of their students, which should also guide the LS that they use to obtain the best academic performance. Expert assistance in the learning process should allow the students to reach their objectives more easily and effectively. Further research could focus on studying PEI and LS along with other variables that influence students and their relationships, along with the effects of these variables on the learning process and academic performance.

Our work also has some limitations. One of these limitations is that this is a transversal study, which does not allow us to establish any causal relationships. Future research should focus on analyzing this relationship with longitudinal studies. Further, in our study, the sample only consists of Spanish students. It therefore might be worth extending the sample to other universities within Europe. Finally, we should note that all the measurements are based on self-reports, which implies the possibility of social desirability bias. Although the perception about emotional capabilities is a key factor for emotional adjustment, it would be interesting to obtain more objective indicators both for EI and LS.

Despite these limitations, this study has shown a relationship between PEI and LS, whilst also distinguishing between the impact of each dimension of the PEI (attention, clarity, and emotional regulation) on each type of LS (cognitive and learning control, support, and studying habits), and showing that there are gender differences in these relationships. These results are relevant for the design of future educational programs focused on the acquisition and development of LS for improving the teaching-learning process between lecturers and students.

## Author Contributions

MV-H, MP-A, and MG-V designed this study; RC and PF-B have conducted it; and all authors (MV-H, MP-A, RC, MG-V, and PF-B) contributed to the research.

## Conflict of Interest Statement

The authors declare that the research was conducted in the absence of any commercial or financial relationships that could be construed as a potential conflict of interest.
